# Assessing Ecological Vulnerability under Climate Change and Anthropogenic Influence in the Yangtze River Estuarine Island-Chongming Island, China

**DOI:** 10.3390/ijerph182111642

**Published:** 2021-11-05

**Authors:** Wanting Peng, Duoduo Wang, Yongli Cai

**Affiliations:** 1 School of Design & China Institute for Urban Governance, Shanghai Jiao Tong University, Shanghai 200240, China; pw2020@sjtu.edu.cn; 2 Shanghai Key Lab for Urban Ecological Processes and Eco-Restoration, School of Ecological and Environmental Sciences, East China Normal University, Shanghai 200241, China; 51153901116@stu.ecnu.edu.cn

**Keywords:** ecological vulnerability, climate change, anthropogenic influence, ecosystem services, urban governance, Yangtze River Estuarine Island

## Abstract

Understanding and assessing ecological vulnerability for estuarine islands are important for maintaining estuarine island ecosystem services and its sustainable development. However, due to its complex fresh water–sea–land interaction mechanism and multiple stressors from both climate change and anthropogenic influence, a comprehensive evaluation of ecological vulnerability for estuarine islands has been limited. Therefore, taking the typical estuary island of Chongming Island as an example, we developed a comprehensive evaluation system of ecological vulnerability for an estuarine island ecosystem based on the pressure-state-response (PSR) conceptual model, and explored the spatial and temporal distribution of ecological vulnerability in 2005 and 2015. The results indicated that the main pressures of Chongming Island from saltwater intrusion intensity and land use intensity were mainly distributed in northern coastal areas and eastern areas of wetland; the ecological vulnerability index (EV) of Chongming Island showed a slight decrease from 2005 to 2015; and three categories of towns based on ecological vulnerability assessment for an eco-island planning and environmental management were identified. Our study provides an effective evaluation system of ecological vulnerability for estuarine islands, which could be helpful for planners and decision makers in improving eco-island planning and environmental management.

## 1. Introduction

As important transition zones between land, freshwater habitats, and the sea, estuaries islands provide many essential ecosystem services [1,2] including coastal blue carbon storage [3], flood protection [4], nutrient cycling, fishery resources [1], and habitats for wildlife [5] as well as offer valuable cultural ecosystem services (e.g., recreation services to citizens for maintaining their mental and physical health) [6,7]. However, with climate change and intensive anthropogenic activities, estuarine islands have been influenced by global sea level rise, ecological structure, and land cover change [8,9], resulting in increasing risks of passive climax submergence, beach soil erosion, biological invasion, and saltwater intrusion [10,11]. These pose as threats to the provision of ecosystem services capacity [12] and maintaining environmental quality as well as human well-being [13].

Estuaries and associated ecosystems, being one of the most vulnerable ecosystems, are delicately affected by both ecological processes of the sea and land and have pressures from multiple anthropogenic stressors and global climate change [1,8]. Climate change is causing a sea level rise, with increasing water temperature and storm frequency [9], which results in estuary coastline retreat, saltwater intrusion, fish and wildlife habitat loss [14], even accelerating its related ecosystem degeneration. In addition, multiple anthropogenic activities also pose as multiple stressors to estuaries. For example, land use and land cover change in estuaries, with the process of transforming natural cover by an impervious surface, have caused shoreline hardening and vegetation loss, which leads to landscape fragmentation, ecological structure, and process change, ultimately altering the ocean and coastal ecosystems [8,11]. Furthermore, an increase in human activities in upstream (i.e., large-scale dam construction) could result in the allocation change of river water resources, reducing the downstream water and causing seawater penetration into the groundwater, all of which pose a serious threat to islands of freshwater resources and coastal ecosystems [15,16]. Furthermore, other increasing human activities including building construction, farmland reclamation, and tourism activities in estuarine islands may have indirect negative effects on introducing exotic species, causing soil compaction and soil pollution through the use of fertilizers and pesticides and other related problems. Together, these multiple stresses make the system vulnerable. However, the mechanism of how these multiple stresses influence the estuary island ecological vulnerability is still unclear. Understanding and assessing ecological vulnerability under climate change and anthropogenic influence on estuarine islands is important [17], and is helpful for planners and decision makers to undertake conservation planning and environmental management for maintaining estuarine island ecosystem services [18], environmental quality and human well-being [19] as well as promoting the estuarine region and the sustainable development of the surrounding urban areas [20].

The concept of ecological vulnerability was originated from ecology theories [21,22] and was first proposed to reflect unpredictable environments. It then was spanned to respond to natural disasters [23] and climate changes [24,25]. Gabor and Griffith (1980) [23] referred to vulnerability as the ability to cope with exposed risks/ threats. Williams and Kapustka (2000) [26] came up with the definition of ecosystem vulnerability accordingly in the symposium held in Seattle, USA [26], which defined it as the potential of an ecosystem to modulate its response to stressors and an estimate of the inability of an ecosystem to tolerate stressors over time and space [17,26]. Until now, many scholars have tried to define ecological vulnerability from different perspectives and hierarchical levels (e.g., organism, population, ecosystem) [17,20,26], but there has been no consistent definition as yet [27]. However, through the exposed stressors, the state and its response to understand and assess ecological vulnerability have been widely recognized [17,20,28]. The comprehensive evaluation based on pressure-state-response (PSR) and its related models (e.g., pressure-state-response-management (PSRM), driver-pressure-state-impact-response (DPSIR) model) have been used in the ecological vulnerability assessment [2,29,30,31]. In recent years, increasing studies of ecological vulnerability evaluation have been widely applied in assessing and identifying ecological problems [25,29], and guiding environmental planning and policies [2,30,32]. Ecological vulnerability evaluation index systems based on the PSRM model and “sensitivity-resilience-pressure” (SRP) model were established to evaluate the spatial and temporal distribution of ecological vulnerability in the Tibet Autonomous Region [27] and Loess Plateau, China [33]. The Pressure–Support–State–Response model (PSSR) was proposed to assess the ecosystem vulnerability of wetland in the Yellow River Delta (a case study of Dongying City) [34], and mountain streams in Azerbaijan [35]. Notwithstanding several calls that have been made for understanding and assessing ecological vulnerability for estuarine islands [36,37], especially those estuarine islands surrounded by urban agglomeration with high anthropogenic influence [38,39], studies on the comprehensive assessment of ecological vulnerability for estuarine islands have been limited due to its complex fresh water–sea–land interaction mechanism and multiple stressors from both climate change and anthropogenic influence [40].

Toward filling this gap, we aimed to better understand the interaction mechanism and develop a comprehensive assessment of ecological vulnerability for estuarine islands. We presented a typical estuarine island—Chongming Island—as an example. It is the largest alluvial island in the world, located off the Shanghai Coast and in the northern part of the estuary of the Yangtze River, which is more vulnerable to climate change caused by sea level rise, storm surge, and salt water invasion [10]. Surround by the urban agglomeration of the Yangtze River Delta (YRD) with intensive urbanization and anthropogenic activity, it is faced with the pressure from anthropogenic activity. It is also the only official eco-island and proposed the world-class eco-island in China, which has been promoted as a plot area of ecological sustainability development [41,42]. Understanding the spatiotemporal distribution of ecological vulnerability of Chongming Island is crucial for policy makers and managers to facilitate planning and promote sustainable development. Therefore, we developed a comprehensive evaluation system of ecological vulnerability for estuarine island ecosystems under climate change and anthropogenic influence. The specific purposes of this study were: (1) to develop a comprehensive evaluation system for estuarine island ecological vulnerability assessments based on PSR conceptual model; and (2) to evaluate the spatial and temporal distribution of the ecological vulnerability of Chongming.

## 2. Materials and Methods

### 2.1. Study Area and Data Sources

**Figure 1:**
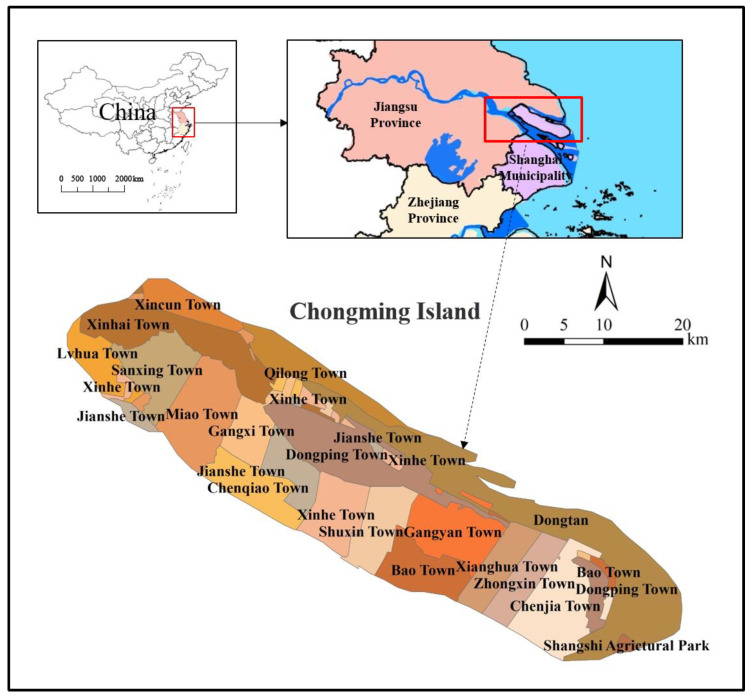
Location of the study area.

Chongming Island (121°09′30″–121°54′00″ E, 31°27′00″–31°51′15″ N) is located on the estuary of the Yangtze River (Figure 1), situated between the East China Sea and the Yangtze River, China. It is one of the world’ s largest estuary alluvial islands, covering an area of 1267 km^2^ with 80 km long from east to west. Chongming Island is known as a biodiversity ‘hotspot’, which is the midpoint of the route of Asia–Australia bird migration and provides habitats for 2–3 million migratory winter birds [43]. The State Council of China is determined to build Chongming Island as the world-class eco-island. However, surround by the urban agglomeration of the YRD, Chongming Island suffers high pressure from anthropogenic activities. By the end of 2019, the total population of Chongming Island was more than 820,000. The high population density has led to conflict between natural conservation and economic development. In addition, as an estuary alluvial island with low elevation (3.21–4.20 m), Chongming Island is affected by sea level rise caused by climate change. Recently, Chongming Island is faced with the main pressure of both climate change and highly anthropogenic influences.

This study collected data from the natural environment, human activities, and management to assess ecological vulnerability from 2000 to 2015, during which Chongming Island has dramatically changed and faced high pressure from climate change and anthropogenic influences. Landsat 30 m spatial resolution data were selected as the imaging time with low cloud cover in the study area in the summer of 2005 and 2015 (http://glovis.usgs.gov/ (accessed on 12 October 2020)) and used to interpret land cover. Land use was interpreted from remote sensing images of Chongming Island in 2005 and 2015, and were categorized according to the secondary classification standard of the Land Use Classification and Code (GB/T 2021010-2017) as follows: urban residential land, river water surface, urban and village road land, tree forest land, paddy field, inland beach, pond water surface, and coastal beach.

Digital elevation model (DEM) data with a spatial resolution of 30 m were downloaded from the China Geospatial Cloud (http://www.gscloud.cn/ (accessed on 18 October 2020)). Rainfall and wind data were obtained from the Baoshan Meteorological Station in Shanghai (No. 58362), whose geographical location is close to Chongming Island and has long-term historical data. Rainfall data in 2005 and 2015 were downloaded with a time resolution of 6 h. Soil salinity was collected from the field soil samples using the sample plots method in November 2016. Soil salt content was measured using the conductivity method. Saltwater intrusion and soil organic matter monitoring data were based on previous studies [44,45], and the nearest resampling methods were used to interpolate saltwater intrusion and soil organics in the study area. Biodiversity data were from the survey report of terrestrial wildlife resources in Shanghai and reclassified land use types using remote sensing data.

### 2.2. The Comprehensive Evaluation System of Ecological Vulnerability

#### 2.2.1. The PSR Evaluation Model

**Figure 2:**
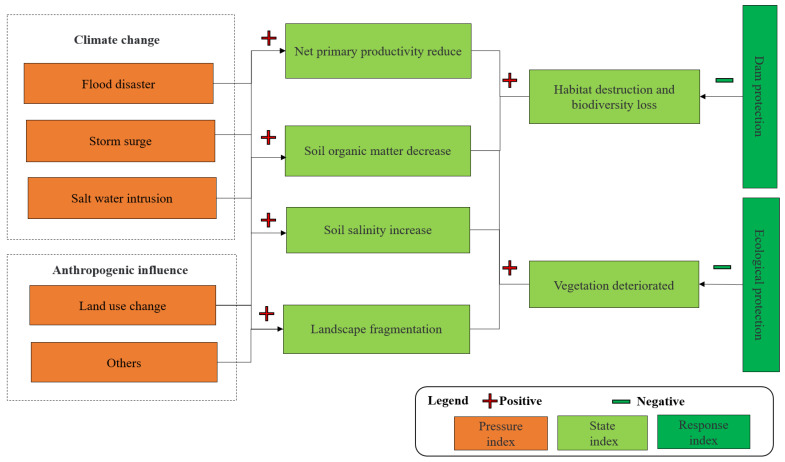
The framework to illustrate the influence factors of ecological vulnerability for estuarine islands.

PSR assessment framework was used to assess the ecological vulnerability of estuarine islands based on the understanding of the fresh water–sea–land interaction mechanism (Figure 2). Estuarine islands, situated between terrestrial ecosystem and sea ecosystem, share similar characteristics and pressures to them. Referring to the relevant research [7,15,46] and combined with field research [47], we identified that flood disaster [4,48], storm surge [49], saltwater intrusion [44], and land use intensity disturbed by human activities [15,50] were the main pressures on estuarine islands. Estuarine islands, located on the low-lying terrain affected by strange airflow and wind [9], are vulnerable to sea level rise caused by climate change and extreme climate. The extreme climate is likely to induce flood disaster, storm surge, and salt water intrusion [51]. For example, climate change caused by extreme climate increased the occurrence of flood disaster and storm surge, which led to a decrease in soil organic matter and the plants’ long-term exposure in water, resulting in vegetation deterioration and habitat destruction. In addition, salt water intruded into estuarine islands, giving rise to shoreline erosion, soil salinization, and vegetation deterioration [47,52], which further affected the coastal ecosystems of islands, exacerbating habitat destruction and biodiversity loss [1,53]. Moreover, the rapid urbanization and increasing human disturbance by transforming natural or semi-natural surface to artificial surface [54] reduces the productivity of vegetation [16] and leads to landscape fragmentation and wildlife habitat destruction [11]. Other human activities including upstream dam construction, farmland reclamation, and tourism activities may increase landscape fragmentation and have a negative impact on vegetation and related ecosystems. Simultaneously, dam protection [55,56] and appropriate protection management [33] can respond to the change of ecological vulnerability by implementing different strategies to natural disasters and man-made disturbances. Finally, 17 indicators were selected from three subsystems (Table 1).

The pressure subsystem consists of four indicators: flood disaster intensity (FDI), storm surge intensity (SSI), saltwater intrusion intensity (SII), and land use intensity (LUI) index (Table 1). FDI indicates the extent and intensity of inundation under the flood peak period, which was calculated by using a rainfall submergence model that combines information on the relationship between altitude, rainfall and inundation depth, the extent, and depth of inundation area (Equation (1)) [15]. We modified rainfall grade standards and pressure intensity in this model according to the multi-year rainfall data of Chongming Island. SSI refers to the damage grade of storm surge along the coastline, which could be represented by the frequency of wind and the wave height of the sea surface. The spatial distribution of SSI is defined by the distance between the island inland and coastline and the storm surge hazard pressure intensity. SSI can be calculated by Equations (2) and (3). Saltwater intrusion intensity (SII) was measured by field samples based on previous study [44]. Land use intensity (LUI) was measured from pressures of different land use types where the LUI coefficient is assigned and modified in each land use [15,31]. The details are listed in Table A1 in Appendix A.

(1) $$I\_Rain_{\left(i,j\right)}=\overset{m}{\underset{n=1}{\sum }}(H\_Rain_{\left(i,j\right)}-H_{\left(i,j\right)})$$

where *I_Rain_(i,j)_* refers to the flood disaster intensity in grid of (*i,j*); *H_Rain_(i,j)_* refers to *n* times of flood disaster intensity in grid of (*i,j*); *H_(i,j)_* refers to the altitude in grid of (*i,j*); and *m* refers to the frequency of different grades of rainstorm.

(2) $$I\_Storm_{\left(i,j\right)}=\sum_{m=1}^{16}\sum_{n=1}^{p}(H_{\max}\_Storm_{\left(i,j\right)}\times Fr_{n})\;$$

where *I_Storm _(i,j)_* refers to the storm surge intensity in grid of (*i,j*); *H_max__Storm _(i,j)_* refers to the maximum wave height caused by a storm surge in grid of (*i,j*); *Fr_n_* refers to frequency of wind force at *n* level; and M refers to wind directions.

(3) $$I\_Storm_{\left(x,y\right)}=\sum_{n=1}^{p}(I\_Storm_{\left(i,j\right)}\times \frac{1}{D})$$

where *I_Storm _(x,y)_* refers to storm intensity in the number of (*x,y*); *I_Storm _(i,j)_* refers to storm intensity in coastal line; and D refers to the distant between grid (*x,y*) to *(i,j).*

There are seven indicators in the state subsystem (Table 1): normalized differential vegetation index (NDVI), enhanced vegetation index (EVI), net primary productivity (NPP), biodiversity indicators (BI), soil organic matter (SOM), soil salinity intensity (SSAI), and landscape fragmentation (LF). NDVI [57] and EVI [58] were extracted from the two scenes in the remote sensing images from 2005 and 2015 in the study area. Soil organic matter content data were obtained from the study by Sun [45], which were measured with the potassium dichromate bulk density method. NPP was used to quantitatively measure the ecosystem’s productive capacity. Biodiversity was considered from a species and landscape level. Biodiversity at the species level index was calculated from the survey report of terrestrial wildlife resources in Shanghai, and the biodiversity at landscape level index was obtained based on land use types. The remote sensing salt inversion method was used to map SSAI in the study area in 2005 and 2015. In order to improve the accuracy of salt inversion, several methods have been used to compare the accuracy and results. The methods included the multivariate adaptive regression splines (MARS) model and partial least squares regression (PLSR) model. In order to reduce the noise and background information of remote sensing image, NDVI, salinity index (SI), SII, and canopy response salinity index (CRSI) were selected as variables to run the salinity inversion model. Given the influence of soil moisture on soil salinity, the land surface water index (LSWI) was calculated as the input of the model. The calculation equations of each index are as follows:

(4) $$\mathrm{NDVI}=(\rho_{\mathrm{NIR}}-\rho_{R})/\left(\rho_{\mathrm{NIR}}+\rho_{R}\right)$$

(5) $$\mathrm{SI}=\sqrt{\rho_{B}{\;*\;\rho }_{R}}$$

(6) $$\mathrm{SI}1=\sqrt{\rho_{G}{\;*\;\rho }_{R}}$$

(7) $$\mathrm{CRSI}=\sqrt{\frac{\rho_{\mathrm{NIR}}{\;*\;\rho }_{R}-\rho_{G}{\;*\;\rho }_{B}}{\rho_{\mathrm{NIR}}{\;*\;\rho }_{R}+\rho_{G}{\;*\;\rho }_{B}}}$$

(8) $$\mathrm{LSWI}=\left(\rho_{\mathrm{NIR}}-\rho_{\mathrm{SWIR}}\right)/\left(\rho_{\mathrm{NIR}}+\rho_{\mathrm{SWIR}}\right)$$

where $\rho_{B}$ refers to the reflectance in blue band of remote sensing data; $\rho_{G}$ refers to the reflectance in the green band of remote sensing data; $\rho_{R}$ refers to the reflectance in the red band of the remote sensing data; $\rho_{\mathrm{NIR}}$ refers to the reflectance in near-infrared band of remote sensing data; and $\rho_{\mathrm{SWIR}}$ refers to the reflectance in short-wave infrared bandⅠ (SWIRI) of the remote sensing data.

LF was measured from the patch scale and landscape scale. Two landscape index at patch scale (i.e., maximum patch index (LPI) and patch density (PD)) were selected to represent the influence degree of the maximum patch on the whole patch and the degree of fragmentation and heterogeneity of the landscape, respectively. Two landscape index of Shannon diversity index (SHDI) and landscape evenness index (SHEI) were used to measure landscape fragmentation at the landscape scale. All of these landscape indexes were calculated in Fragstats 4.2 software [59,60], and these equations are as follows.

(9) $$\mathrm{PN}=\frac{n_{i}}{A}$$

where *n_i_* refers to the total area of landscape elements in *i*th and A refers to the total area of all landscape.

(10) $$\mathrm{SHDI}=-\overset{m}{\underset{i=1}{\sum }}\left(p_{i}\ln p_{i}\right)$$

(11) $$\mathrm{SHEI}=\frac{-\sum_{i=1}^{m}\left(p_{i}\ln p_{i}\right)}{\ln m}$$

where *p_i_* refers to the ratio occupied by landscape patch type *i* and *m* refers to the total number of landscape patches.

Two indicators of ecological protection grades (EPG) and dam protection capabilities (DPC) were selected in the response subsystem. EPG were reclassified as the level of government’ s supervision and protection intensity in different regions according to the “General Plan of Chongming District of Shanghai and General Plan of Land Use (2016–2040)”. The previously collected data in 2007 [15] and field survey data in 2015 were used to digitize the dam protection data of Chongming Island. Since the northern dam is an earth dam, the protection grade is 0.5 whereas the southern dam is a cement dam, which has a value of 1.

#### 2.2.2. The Weight of Indicators

The weight of each indicator was determined by the spatial principal component analysis (SPCA) method. The SPCA method has shown good performance in classifying the differences among the data and avoiding the spatial correlation by reducing the dimensionality of a dataset while retaining as much information as possible, which has been widely used in index weight calculations [34]. The principal components with a cumulative contribution rate greater than 85% in 2005 and 2015 were extracted, and the weight of each indicator for the ecological vulnerability assessment is shown in Table A2; Table A3; Table A4. The calculation equation of ecological vulnerability value for each pressure, state, and response subsystem is as follows:

(12) $$L=r_{1}P_{1}+r_{2}P_{2}+r_{3}P_{3}+\ldots +r_{n}P_{n}$$

where *L* refers to ecological vulnerability value for each pressure, state, response subsystem; *P_i_* refers to the *i*th principal component; and *r_i_* refers to the contribution rate corresponding to the *i*th principal component.

#### 2.2.3. Ecological Vulnerability Evaluation

The ecological vulnerability (EV) index was used to indicate the degree of the ecological vulnerability of estuarine islands (Equation (13)). EV was measured using pressure, state, and response variables [33]. As defined by normalized ecological vulnerability index (Equation (14)), we categorized the ecological vulnerability for estuarine islands into five classes: (1) extremely vulnerable, EV > 0.8; (2) high vulnerable, 0.6 < EV ≤ 0.8; (3) moderately vulnerable, 0.4 < EV ≤ 0.6; (4) marginally vulnerable, 0.2 < EV ≤ 0.4; and (5) slightly vulnerable, EV ≤ 0.2 [27,61]. Then, we used K-means cluster analysis to identify the towns’ groupings for management according to their normalized ecological vulnerability values. The K-means cluster analysis was run with 1000 iterations to maximally decrease the total error sum of squares (TESS) among clusters [62]. We used ArcGIS 10.6 [63] for SPCA and mapping of ecological vulnerability and R software for K-means cluster analysis.

(13) EV=P+S−R

where *EV* refers to ecological vulnerability index; *P* refers to the pressure; *S* refers to the state; and *R* refers to the response.

(14) $$NEV=\frac{X_{i}-X_{\min}}{X_{\max}-X_{\min}}$$

where *NEV* refers to normalized *EV*; *X_i_* refers to *EV* in *i*; *X*_max_ refers to the maximum *EV* value; and *X*_min_ refers to the minimum *EV* value.

## 3. Results

### 3.1. Spatiotemporal Distribution of EV in PSR Subsystems

**Figure 3:**
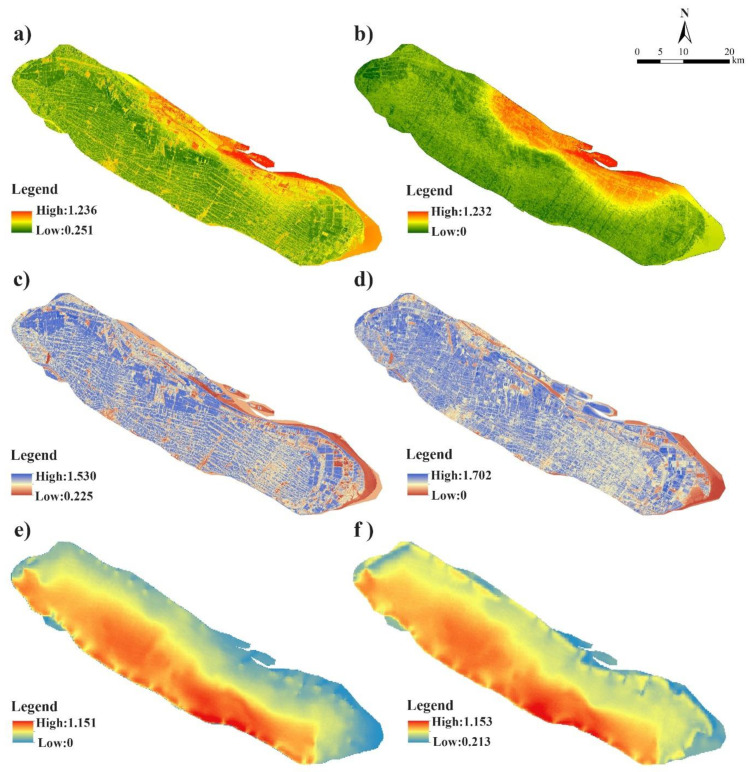
The ecological vulnerability assessment results of the three subsystems of Chongming Island in 2010 and 2015. (**a**) shows the result of pressure subsystem in 2005; (**b**) shows the result of pressure subsystem in 2015; (**c**) shows the result of the state subsystem in 2005; (**d**) shows the result of the state subsystem in 2015; (**e**) shows the result of the response subsystem in 2005; (**f**) shows the result of the response subsystem in 2015.

The results showed the overall mean value of pressure in Chongming Island was higher in 2015 (0.659) than in 2005 (0.606), which were mainly influenced by SII and FDI (Table A2). The high values in the pressure layer were mainly distributed in the northern coastal area and eastern wetland area in 2005 (Figure 3), while the high pressure areas were concentrated and expanded in the northern area. In contrast, the northern coastal area and eastern wetland area achieved low values in the state layer. The overall value of state in Chongming Island had a significant increase from 2005 (0.775) to 2015 (1.075), which benefited from the large increase in natural vegetation and decrease in soil salinization risk with restoration and conservation projects. The response in Chongming was mainly determined by DPC and EPG. The high value response of areas was in the southern and central areas of Chongming Island, while low value areas were along the north and Dongtan area due to the low protection grade of the dam. The distribution of response in Chongming Island showed a similar trend, and the overall mean value of the response layer in Chongming Island experienced a slight increase from 0.649 in 2005 to 0.708 in 2015.

### 3.2. Spatiotemporal Distribution of the Overall EV

**Figure 4:**
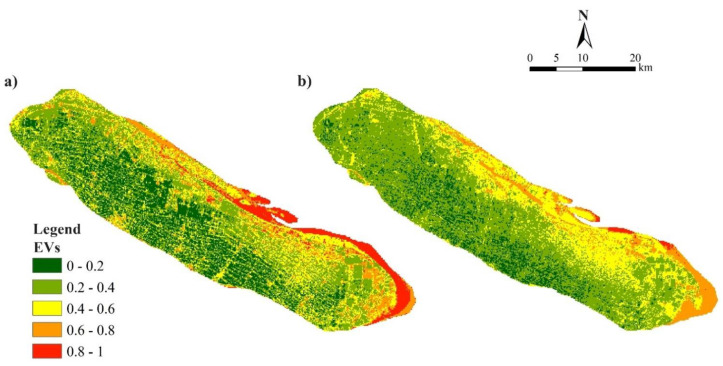
The overall ecological vulnerability assessment results of Chongming Island in 2010 (**a**) and 2015 (**b**).

The EV result of Chongming Island showed a decreasing trend from 0.39 in 2005 to 0.36 in 2015 (Figure 4), with a significant difference in spatial and temporal pattern. From 2005 to 2015, the slightly vulnerable EV (<0.2) and higher EV areas (>0.6) were transformed into moderately and marginally vulnerable levels of EV (0.2–0.6) (Table 2). Some forest lands were converted into agricultural land and construction land, while in the northern area, due to sediment accumulation, the areas that were directly inundated with seawater were converted into wetlands, and even forest land. Spatially, Chongming Dongtan and Qilong Town had the highest EV due to the effects of saltwater intrusion and flooding; Xincun Township and Shangshi Agricultural Park had relatively higher EV, while the remaining regions had relatively lower EV.

### 3.3. EV in Towns and Its Clusters

**Figure 5:**
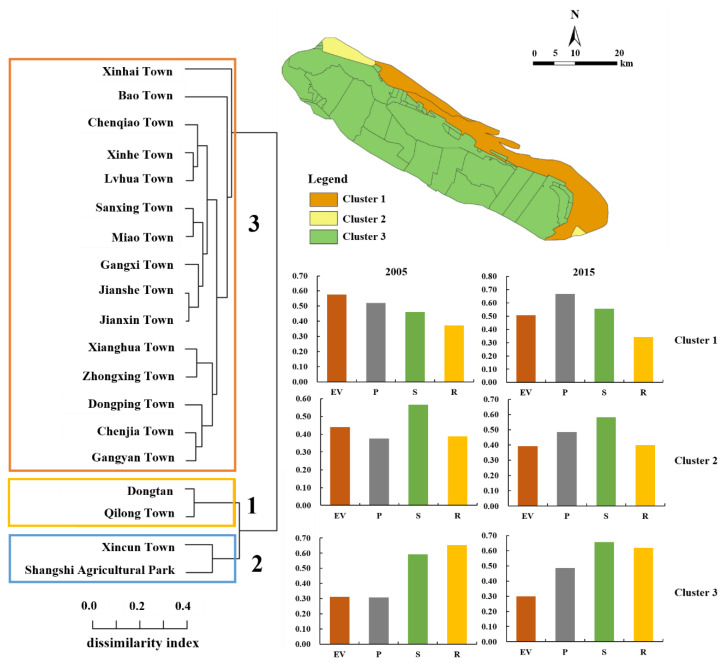
The results of the Chongming Island town clusters based on ecological vulnerability assessment in 2010 and 2015.

The results of cluster analysis indicated EV of Chongming Island in 2005 and 2015 were classified into three categories (Figure 5; Table A5 and Table A6). The first category was the area with high EV, which were mainly distributed in Dongtan and Qilong Town; the second category was the area with moderate ecological vulnerability (e.g., Xincun Town and Shangshi Agricultural Park); and the third category was mainly the remaining towns with relatively low EV.

## 4. Discussion

Our results demonstrated that the comprehensive evaluation system based on the PSR conceptual model could be an effective approach to assess the ecological vulnerability of estuarine islands. Unlike studies from Wagner and Sallema-Mtui (2016) [46], which used repeated quantitative ecological plot and rapid assessment to assess vulnerability in the Rufiji Estuary, Tanzania, our approach has the advantages of assessing an estuarine island system with a more comprehensive perspective, which considers multiple stresses from climate change (e.g., storm surge, salt water invasion, and human influences combined with multiple state and response indicators). In addition, our method could provide spatially explicit information on ecological vulnerability. More importantly, by proposing a comprehensive evaluation system, the spatiotemporal distribution of EV can be easily understood from three dimensions of PSR, which could be helpful for managers and decision makers to facilitate conservation planning and environmental management.

Our results indicated that LUI, SII, NDVI, and DPC were identified as the main driving factors of pressure–state–response subsystems for ecological vulnerability of estuarine island by using SPCA. A similar finding was also found in Tong and Yongbo (2014) [64], which found that land use, influenced by increased population and economic development in the last decade, was the main pressure for Chongming Island. Saltwater intrusion is one of the most serious threat to the estuarine island ecosystem, which can be understood from a knock-on effect on ecological stress [44]. The push of tidewater and surface circulation results in upstream movement of the salt tide in both the southern and northern branches of the Yangtze River. In addition, with the increasing dam constructions in the upper reaches of the Yangtze River [16], the freshwater reduced dramatically and exacerbated saltwater intrusion. As a result of the alluvial silt soil properties of the islands, which is closely linked to the groundwater and the water of the sea, the sea island moves by tidal action into the groundwater system and the aquifer rapidly spreads its stress, resulting in soil salinization and a crisis of freshwater on the island [50], which showed a negative knock-on effect on terrestrial plants, coastal, and wetland ecosystems [51,65]. It is worth noting that the pressures on Chongming Island were largely caused by climate change and human activities (e.g., intrusion of salt water), which may gradually threaten the ecosystem security of Chongming Island in the future. Vegetation related indicators (i.e., NDVI and EVI) could indicate the state of the ecosystem by representing the growth status of island vegetation under external disturbance, which is consistent with the results of previous studies [34,66]. Under the influence of human activities, the vegetation index decreases in the area near settlements. In terms of response, ecological protection grades and dam protection capabilities could reflect the spatial heterogeneity and intensity of response. In previous studies, human protection data and management were limitedly used to indicate response subsystem, but the results of this paper showed that ecological protection grades had good performance in indicating response to ecological vulnerability [33].

Understanding the spatiotemporal distribution of ecological vulnerability under climate change and the anthropogenic influence on estuarine islands is important to help decision makers undertake conservation planning and environmental management [17]. The spatial and temporal distribution of EV from 2005 and 2015 were revealed in our study. Our results suggest that the area of high EV decreased during the period of 2005–2015 with effective restoration projects and protected areas construction (e.g., Dongtan National Wetland Park) [67]. The decrease in ecological vulnerability in Chongming Island can be explained by the effective restoration projects and construction of protected areas (e.g., Shanghai Chongming Dongtan National Nature Reserve) to increase natural vegetation and mitigate ecosystem degradation. For example, the Shanghai Chongming Dongtan National Nature Reserve was established in 2005, and strict protection management with wetland restoration has been implemented for protecting wetlands and other habitats for wildlife and birds in the East Asian–Australasian bird migration route [53,67]. With cluster analysis, we found three categories of towns in Chongming Island in both 2005 and 2015, which could be helpful for decision makers to develop specific strategies and improve environmental management [17]. We found that the cluster 1 area, on the east beach and the north edge of Chongming Island, which were frequently influenced by storm surge, salt water intrusion, and flood disaster, had a higher ecological pressure with poor condition of vegetation status, which achieved a higher EV, although it had a better protection level. We found that the cluster 2 area on the east beach and the north edge of Chongming Island was frequently influenced by storm surge, saltwater intrusion, and flood disaster. The cluster 2 area had a higher ecological pressure with poor condition of vegetation status, which achieved a higher EV, although it had a better protection level. Areas of cluster 3 were located in the south coast and middle of the island and they had relatively less pressure, higher state, and response level with the low EV.

Since 2005, the concept of ‘‘Eco-island’’ was proposed in the Master Development Plan for Chongming Island, and in 2010, the plan of “The outline of Chongming Eco-island (2010–2020)’ was issued to construct a world class eco-island as a main goal for the future development of Chongming Island [43,67]. A series of natural ecosystem protection management has been implemented such as the establishment of protected areas, wetland restoration, and strict land resource management [43], and further implications for eco-island management are in great need. Specific implications were put forward to each town bundle based on an ecological vulnerability assessment for the future development Chongming Eco-Island. As for towns in cluster 1 (i.e., Dongtan and Qiming Town), it is necessary to maintain the upstream inflow fresh water volume to prevent saline water invasion; meanwhile, physical methods (e.g., building filtration layer and sluice) can be used to mitigate the negative influence from salt water intrusion. Saline–alkaline tolerance plants are encouraged to be introduced in coastal areas to rebuild and rehabilitate wetlands. The rational extension of the existing embankment should be carried out to increase its protection area, and the poor-quality dikes should be fixed and rebuilt. As for towns in cluster 2 (i.e., Xincun town and Shangshi Agricultural Park), strategies related to the state and response subsystem should be strengthened. Saline–alkali tolerant of vegetation or crops can be selected, and the soil in the Shanghai Agricultural Park can be desalted by physical methods. Similar to cluster 1 towns, extension of the embankment should be carried out to protect the area as much as possible. Meanwhile, unqualified embankment should be reinforced and repaired. As for the towns in cluster 3, with relatively low EV, monitoring and early-warning systems are encouraged to be established to prevent and mitigate disasters caused by climate change and human influence. Moreover, the public participation should be encouraged to improve their awareness of disaster prevention. To avoid vegetation deterioration, we encourage the protection and introduction of native plants. The core zone of protection should take strict protection measurements to prevent high conservation value areas from being land occupation. Our study was, however, limited to one single case of a Yangtze River estuarine island. Future work should apply this comprehensive evaluation system of ecological vulnerability in other estuarine islands. With the development of the Chongming Eco-Island and other estuarine islands, assessment of the ecological vulnerability is of great need, which could be applied to facilitate eco-island planning and management, and promoting sustainable development on estuarine islands.

## 5. Conclusions

This study presents a comprehensive evaluation system to assess the ecological vulnerability of estuarine islands by considering the interaction mechanism between the sea and river, which could correspond better to multiple stressors from both climate change and anthropogenic influence. This is a pioneer study to develop an evaluation index system and to explore the spatial and temporal distribution of ecological vulnerability of Chongming Island under climate change and anthropogenic influence.

Our results showed that saltwater intrusion and land use change had become one of the most significant pressures on the sustainability of Chongming Island. Necessary strategies to mitigate saltwater intrusion and land use change under climate change and human influences should be taken. Ecological vulnerability decreased from 2005 to 2015 with effective restoration projects and the construction of protected areas. Our results suggest that these protection managements should be encouraged. Our study could not only provide implications for the planning and environmental management of the eco-island of Chongming Island, it could also shed light on other estuarine islands, especially these estuarines surrounded by urban agglomeration, to maintain the provision capacity of ecosystem services, environmental quality, and human well-being, and promote the estuarine region and the sustainable development of the surrounding urban areas [20].

## Figures and Tables

**Table 1:** The selected indicators of the estuarine islands’ ecological vulnerability assessment index system [57,58,59,60].

Criterion Layer	Indicator	Meaning	Positive/Negative
Pressure	Flood disaster intensity (FDI)	The extent and intensity of inundation under flood peak period.	+
	Storm surge intensity (SSI)	The damage grade of storm surge along the coastline.	+
	Saltwater intrusion intensity (SII)	The extent and intensity influence by storm surge under storm surge.	+
	Land use intensity (lui)	The pressure of ecosystem caused by the change of land cover caused by human activities.	+
State	Normalized differential vegetation index (ndvi)	NDVI indicates the state of vegetation with low vegetation cover.	−
	Enhanced vegetation index (evi)	EVI indicates the state of vegetation with high vegetation cover.	−
	Net primary productivity (npp)	It indicates the degree of influence of external disturbance on ecosystem productivity.	−
	Landscape fragmentation (lf)	Represents the state of the ecosystem under the interference of human activities at both patch and landscape level.	+
	Biodiversity indicators (bi)	BI indicates the state of biodiversity under human influence.	−
	Soil organic matter (som)	Indicates the state of the physical and chemical properties of soil under the intrusion of salt water.	−
	Soil salinity intensity (SSAI)	Under the intrusion of seawater into groundwater and human intervention, under the action of strong evaporation and low rainfall washing, it represents the damaged state of soil	+
Response	Ecological protection grades (EPG)	Indicates the effective managements respond to climate change and human influence.	−
	Dam protection capabilities (DPC)	Indicates humans actions are responding to storm surge and sea level rise.	−

**Table 2:** The statistics of the ecological vulnerability assessment results of Chongming Island in 2005 and 2015.

EV	Class	Area in 2005 (%)	Area in 2015 (%)
<0.2	Slightly vulnerable	66.87	56.20
0.2–0.4	Marginally vulnerable	10.07	23.12
0.4–0.6	Moderately vulnerable	7.62	14.44
0.6–0.8	High vulnerable	7.51	4.63
>0.8	Extremely vulnerable	7.93	1.60

## Data Availability

Publicly available datasets were analyzed in this study. These data can be found here: USGS Global Visualization Viewer (GloVis) (http://glovis.usgs.gov/ (accessed on 12 October 2020)); China Geospatial Cloud (http://www.gscloud.cn/ (accessed on 18 October 2020)).

## Appendix A

**Table A1:** Classification of land use intensity.

Classes	LULC	Feature	*p* Value
I	Salt marshes, ocean	Least affected by human activity with low LUI and P value	1
II	Inland marshes	Partly influenced by human activity, with relatively low LUI	2
III	Forest, river	Moderate influenced by human activity, with moderate LUI and P value	3
IV	Paddy fields, pond	High influenced by human activity, with moderate LUI and P value	4
V	Urban land, Country residential land, Other build-up land	Extremely high with moderate LUI, and land cover changes irreversibly, with the highest LUI	5

**Table A2:** Eigenvalues and correlation coefficient matrix of principal components of pressure indicators in 2005 and 2015.

	2005			2015		
	PC1	PC2	PC3	PC1	PC2	PC3
Eigenvalue (λ)	0.05	0.02	0.02	0.04	0.03	0.01
Contribution rate (%)	51.62	22.15	19.13	50.66	38.17	10.82
Cumulative contribution rate (%)	51.62	73.77	92.90	50.66	88.83	99.64
LUI	0.32	0.34	0.88	0.13	0.99	−0.08
FDI	0.30	0.82	−0.44	0.01	0.00	−0.01
SSI	0.28	0.07	−0.07	0.24	0.04	0.97
SII	0.85	−0.44	−0.15	0.96	−0.14	−0.24

**Table A3:** Eigenvalues and correlation coefficient matrix of principal components of state indicators in 2005 and 2015.

	2005			2015		
	PC1	PC2	PC3	PC1	PC2	PC3
Eigenvalue (λ)	0.06	0.02	0.02	0.06	0.04	0.01
Contribution rate (%)	54.40	18.04	14.50	43.73	28.79	14.38
Cumulative contribution rate (%)	54.40	72.44	86.94	43.73	72.52	86.90
SSAI	−0.10	−0.02	−0.01	−0.21	−0.33	−0.06
NDVI	0.66	−0.01	0.01	0.46	0.40	−0.10
EVI	0.67	0.04	−0.03	0.49	0.44	−0.11
LI	0.00	0.16	0.99	0.00	0.03	−0.24
BI	0.03	0.89	−0.14	−0.68	0.72	0.09
SOM	0.17	−0.42	0.08	0.15	0.01	0.95
NPP	0.27	0.06	0.01	0.13	0.12	0.05

**Table A4:** Eigenvalues and correlation coefficient matrix of principal components of response indicators in 2005 and 2015.

	2005		2015	
	PC1	PC2	PC1	PC2
Eigenvalue (λ)	0.03	0.01	0.02	0.01
Contribution rate (%)	75.04	24.96	72.04	27.96
Cumulative contribution rate (%)	75.04	100.00	72.04	100.00
EPG	0.93	0.36	0.92	0.40
DPC	−0.36	0.93	−0.40	0.92

**Table A5:** The ecological vulnerability of each town in 2005.

Town	EV	SSI	FDI	SII	LUI	SSAI	NDVI	EVI	LI	BI	SOM	NPP	EPG	DPC
Dongtan	0.588	0.328	0.382	0.550	0.400	0.133	0.471	0.498	0.732	0.253	0.158	0.139	0.485	0.291
Qilong Town	0.566	0.296	0.162	0.609	0.430	0.162	0.465	0.437	0.733	0.320	0.312	0.129	0.401	0.272
Xincun Town	0.425	0.418	0.380	0.105	0.366	0.099	0.671	0.569	0.775	0.208	0.293	0.204	0.376	0.283
Chenjia Town	0.342	0.108	0.370	0.108	0.410	0.117	0.661	0.513	0.789	0.178	0.339	0.203	0.196	0.678
Sanxing Town	0.272	0.045	0.419	0.040	0.354	0.102	0.742	0.669	0.768	0.203	0.273	0.241	0.391	0.726
Xinhai Town	0.331	0.173	0.205	0.081	0.366	0.103	0.724	0.672	0.784	0.214	0.367	0.249	0.377	0.415
Miao Town	0.273	0.092	0.361	0.036	0.367	0.105	0.721	0.646	0.776	0.195	0.369	0.232	0.389	0.727
Chenqiao Town	0.317	0.175	0.351	0.046	0.479	0.127	0.621	0.543	0.785	0.158	0.508	0.187	0.274	0.762
Gangxi Town	0.258	0.021	0.374	0.065	0.377	0.109	0.709	0.646	0.758	0.186	0.591	0.223	0.377	0.726
Jianshe Town	0.288	0.068	0.374	0.069	0.425	0.117	0.674	0.618	0.766	0.227	0.496	0.214	0.393	0.705
Dongping Town	0.322	0.027	0.216	0.257	0.371	0.104	0.719	0.615	0.767	0.205	0.351	0.241	0.368	0.548
Xinhe Town	0.319	0.104	0.386	0.129	0.428	0.120	0.652	0.603	0.759	0.166	0.370	0.200	0.362	0.764
Shuxin Town	0.288	0.080	0.340	0.064	0.420	0.118	0.680	0.597	0.767	0.176	0.574	0.211	0.395	0.679
Bao Town	0.253	0.151	0.270	0.059	0.445	0.123	0.644	0.540	0.753	0.194	0.762	0.199	0.362	0.800
Gangyan Town	0.341	0.033	0.260	0.215	0.418	0.119	0.692	0.551	0.774	0.165	0.438	0.215	0.390	0.546
Xianghua Town	0.367	0.091	0.307	0.299	0.456	0.130	0.643	0.517	0.770	0.140	0.507	0.189	0.379	0.611
Zhongxing Town	0.397	0.073	0.345	0.267	0.462	0.133	0.621	0.504	0.786	0.125	0.413	0.176	0.391	0.618
Shangshi Agricultural Park	0.455	0.586	0.575	0.049	0.456	0.120	0.566	0.440	0.753	0.260	0.259	0.179	0.533	0.275
Lvhua Town	0.328	0.217	0.439	0.030	0.398	0.115	0.646	0.595	0.762	0.173	0.237	0.185	0.373	0.806

**Table A6:** The ecological vulnerability of each town in 2015.

Town	EV	SSI	FDI	SII	LUI	SSAI	NDVI	EVI	LI	BI	SOM	NPP	EPG	DPC
Dongtan	0.508	0.315	0.495	0.550	0.401	0.505	0.551	0.423	0.811	0.441	0.158	0.139	0.485	0.420
Qilong Town	0.509	0.331	0.255	0.609	0.460	0.487	0.470	0.422	0.816	0.407	0.312	0.129	0.401	0.387
Xincun Town	0.379	0.283	0.536	0.105	0.415	0.440	0.617	0.646	0.823	0.372	0.293	0.204	0.376	0.442
Chenjia Town	0.344	0.187	0.519	0.108	0.427	0.448	0.580	0.592	0.807	0.378	0.339	0.203	0.196	0.730
Sanxing Town	0.273	0.121	0.583	0.040	0.407	0.415	0.696	0.698	0.815	0.258	0.273	0.241	0.391	0.786
Xinhai Town	0.308	0.259	0.323	0.081	0.338	0.446	0.698	0.699	0.828	0.301	0.367	0.249	0.377	0.479
Miao Town	0.265	0.221	0.516	0.036	0.453	0.426	0.670	0.676	0.822	0.250	0.369	0.232	0.389	0.779
Chenqiao Town	0.270	0.249	0.500	0.046	0.566	0.454	0.576	0.585	0.763	0.236	0.508	0.187	0.274	0.809
Gangxi Town	0.252	0.039	0.536	0.065	0.423	0.416	0.679	0.659	0.805	0.265	0.591	0.223	0.377	0.767
Jianshe Town	0.272	0.179	0.530	0.069	0.426	0.425	0.657	0.629	0.811	0.270	0.496	0.214	0.393	0.736
Dongping Town	0.347	0.013	0.341	0.257	0.344	0.443	0.666	0.687	0.814	0.353	0.351	0.241	0.368	0.590
Xinhe Town	0.287	0.248	0.546	0.129	0.436	0.425	0.646	0.608	0.805	0.272	0.370	0.200	0.362	0.806
Shuxin Town	0.262	0.197	0.485	0.064	0.455	0.400	0.641	0.638	0.812	0.309	0.574	0.211	0.395	0.745
Bao Town	0.235	0.191	0.408	0.059	0.506	0.442	0.586	0.602	0.800	0.290	0.762	0.199	0.362	0.836
Gangyan Town	0.362	0.018	0.397	0.215	0.491	0.398	0.597	0.637	0.818	0.405	0.438	0.215	0.390	0.573
Xianghua Town	0.364	0.178	0.441	0.299	0.497	0.405	0.568	0.573	0.815	0.444	0.507	0.189	0.379	0.644
Zhongxing Town	0.375	0.172	0.488	0.267	0.467	0.406	0.567	0.541	0.829	0.463	0.413	0.176	0.391	0.667
Shangshi Agricultural Park	0.408	0.295	0.665	0.049	0.413	0.593	0.496	0.508	0.802	0.435	0.259	0.179	0.533	0.482
Lvhua Town	0.286	0.234	0.600	0.030	0.475	0.467	0.627	0.581	0.811	0.257	0.237	0.185	0.373	0.794
